# Drug Companies Should Be Held More Accountable for Their Human Rights Responsibilities

**DOI:** 10.1371/journal.pmed.1000344

**Published:** 2010-09-28

**Authors:** The PLoS Medicine Editors

## Abstract

The *PLoS Medicine* Editors argue that drug companies should be held more accountable for their human rights responsibilities.

Almost ten years ago, activism and public protest resulted in a landmark victory for the access to medicines movement, when 39 of the world's leading pharmaceutical companies dropped their collective lawsuit against the South African government for attempting to legalize the importation of cheaper generic versions of drugs [1]. Following a settlement, medication prices—including those for antiretrovirals to treat the millions of South Africans with HIV/AIDS—dropped, and public pressure on the pharmaceutical industry was hailed a success [2]. A decade later it's time again for action—to hold drug companies accountable for their human rights responsibilities to make medicines available and accessible to those in need.

As part of this effort, *PLoS Medicine* publishes three unique perspectives on the question of whether drug companies are living up to their human rights responsibilities. Sofia Gruskin and Zyde Raad from the Harvard School of Public Health say more assessment is needed of such obligations [3]; Geralyn Ritter, Vice President of Global Health Policy and Corporate Social Responsibility at Merck & Co., argues that multiple stakeholders could do more to help States deliver the right to health [4]; and Paul Hunt and Rajat Khosla offer their reflections [5] on Mr. Hunt's work as the UN Special Rapporteur on the right to the highest attainable state of health (2002–2008), which culminated in the first “Human Rights Guidelines for Pharmaceutical Companies” [6]. This *PLoS Medicine* Debate comes two years after the release of the Guidelines and a year after Mr. Hunt's report [7] on his invited mission to review the policies and practices of GlaxoSmithKline (GSK) was submitted to the UN Human Rights Council. Together these perspectives and reports make clear that the responsibilities of pharmaceutical companies go beyond stakeholder value to encompass human rights. What is also clear is that more accountability is now needed.

The Guidelines on human rights responsibilities of drug companies—based upon an extensive and consultative process with multiple stakeholders—include responsibilities for transparency, management, monitoring and accountability, pricing, and ethical marketing. They identify best practice as including access to medicines, differential pricing between and within countries, commercial voluntary licensing that allows for the production of cheaper generics, investment into research and development (R&D) of neglected diseases, and engagement in public–private partnerships. The Guidelines also recommend against lobbying for more protection in intellectual property laws, applying for patents for trivial modifications of existing medicines, inappropriate drug promotion, and excessive pricing. Indeed, a most notable aspect of the Hunt Report is its revelation that many of the obstacles States face in delivering the right to health to their citizens are created by these very drug company practices [7].

The chief proposition in the Guidelines is that pharmaceutical companies, by virtue of being granted by society the monopoly and power to develop medicines, hold that power (the patent) with express conditions. And these conditions include human rights responsibilities to make medicines available and accessible. As Hans Hogerzeil, WHO Director of Essential Medicines and Pharmaceutical Policies, was quoted recently as saying, not providing new drugs available to those who need them “breaks the social contract and works against the duty to promote human rights—as the company that holds the patent is basically the only entity that can legally do so” [8]. At the same time that the 825 billion dollar global pharmaceutical industry operates as society's chief developer and purveyor of life-saving medicine, two billion people around the world lack access to essential medicines. Such a persistent perversity demands more outrage.

Unfortunately, the pharmaceutical companies that now do acknowledge the importance of principles related to human rights tend to blunt their own responsibilities by instead emphasizing their corporate social responsibility initiatives, employment standards, global health programmes, and participation in drug donation schemes, voluntary price reductions, or international business groups like the UN Global Compact and the Business Leaders Initiative on Human Rights (BLLIHR) [9]–[12]—not explicitly human rights activities. Indeed, there is danger in pharmaceutical companies' persistent assertions that the primary responsibility for delivering the right to health lies with the State and that their role is merely supportive—as reflected in the Merck perspective published today in *PLoS Medicine* [4]—as this argument allows the industry to exculpate itself from its own human rights responsibilities.

Klaus Leisberger, President of the Novartis Foundation for Sustainable Development, is a leading advocate of corporate social responsibility among pharmaceutical companies, but nevertheless writes that “no private enterprise has the societal mandate or the organizational capabilities to feed the poor or provide health care to the sick” [13]. “Enlightened” pharmaceutical companies, he says, can respect and even protect the right to health of their employees, for example. But beyond that, in terms of delivering the right to health, he argues that this responsibility “cannot be more than a very limited contribution to overcoming the challenges that we all face on a global level.” And indeed major companies in their responses to the Guidelines argued that their role and human rights responsibilities are not adequately defined [14],[15]. In contrast, independent observers have stated that these expectations are in fact well delineated [16].

So how well are drug companies doing? Several exercises are available that shed some light on the question. The most important of these, the Access to Medicines Index (http://www.accesstomedicineindex.org/), provides an unprecedented and independent comparative analysis of 27 top drug companies on measures of commitments, performance, transparency, and R&D in relation to access to medicines. Its 2010 Report ranks companies in several areas and details improvements since 2008—this year GSK topped the list again, and appears to be a consistently viewed industry leader. Merck & Co. and Novartis were ranked two and three in 2010, with Pfizer and Gilead emerging as the most improved since 2008. The Index concludes that many more companies are participating in initiatives to improve access to medicines and are becoming more transparent and cooperative in sharing information but that the need for medicines across a range of infectious and non-communicable diseases remain substantial and is growing. (Notably, the Index does not examine company performance against best practices, but instead against other companies.)

Other analyses, by the George Institute, the UK Department of International Development, and others [5] demonstrate some progress in access to medicines and many areas where improvement is needed. But none of these audits, including the Access to Medicine Index, “frame the performance indicators in human rights terms” and none “explicitly advocate a requirement for accountability against human rights standards,” emphasised Paul Hunt in an interview with *PLoS Medicine*.

What type of accountability is needed?

The importance and significance of accountability in this area cannot be overstated. As Helen Potts argues, accountability is not merely “responsiveness, responsibility, answerability or evaluation” [17]. Where the pharmaceutical industry and access to medicines intersect, accountability is something much more than that and must go to the core of the business activity. Paul Hunt's work [5] highlights the critical step of companies creating independent, transparent mechanisms to monitor and publicly document their own activities. We agree. Beyond an add-on or peripheral activity, the acknowledgement and promotion of human rights must become a regular, integrated aspect of the work of pharmaceutical companies. Better yet would also be an external, international body charged explicitly with monitoring the policies and practices of pharmaceutical companies and reporting publicly on the discharge of their right-to-health responsibilities.

The challenge for the pharmaceutical industry is to develop viable business models that allow for profit whilst respecting and promoting human rights. Pharmaceutical companies are tremendously innovative entities that abhor bad publicity, so the incentive is there for stimulating creative thinking under public pressure. The human rights guidelines and responsibilities are now clear; the monitoring and accountability must step up in earnest.

## Abbreviations

GSK: GlaxoSmithKline
R&D: research and development
